# A multicenter point-prevalence survey of antibiotic utilization patterns in Ethiopia: implications for strengthening stewardship programs

**DOI:** 10.1186/s12879-026-12775-z

**Published:** 2026-02-03

**Authors:** Mekonnen Damessa, Desta Assefa, Gemmechu Hasen, Belachew Umeta, Tsegaye Melaku, Geremew Kinati, Kedir Abdella Abdulsemed, Dereje Kebede, Habtewold Deti Waktola, Esayas Kebede Gudina, Sultan Suleman

**Affiliations:** 1 Jimma University Laboratory of Drug Quality (JuLaDQ), Jimma, Oromia Ethiopia; 2 School of Pharmacy, Jimma University, Jimma, Oromia Ethiopia; 3 Clinical Trials Directorate, Armauer Hansen Research Institute, Addis Ababa, Ethiopia; 4 Department of Pharmacy, Wollaga Univiversity, Nekemte, Oromia Ethiopia; 5 Department of Medical Microbiology, School of Medical Laboratory, Jimma University, Jimma, Oromia Ethiopia; 6 Department of Pharmacy, Adama Science and Technology University, Adama, Oromia Ethiopia; 7 Department of Internal Medicine, School of Medicine, Jimma University, Jimma, Oromia Ethiopia

**Keywords:** Antibiotic utilization, Empirical prescribing, Point prevalence survey, Antimicrobial resistance, Ethiopia

## Abstract

**Background:**

The emerging of antimicrobial resistant strains is destroying the paradigm-shifting power of antibiotics. The main driver of antimicrobial resistance inappropriate prescribing practices, primarily in institutions like Sub-Saharan Africa. Hence, this study aims to evaluate real antibiotic use patterns among inpatients at four public hospitals in Jimma, Ethiopia.

**Methods:**

Across-sectional point prevalence survey using standardized World Health Organization (WHO)’s point-prevalence survey (PPS) was employed to conduct this study in August 2023. All hospitalized patients at adult and pediatric wards before or at 08:00 on the survey date were enrolled. Data collection tool was adopted and customized to collect hospital antibiotic use pattern using a standardized PPS method. Data were collected using Open Data Kit (ODK) and analyzed using SPSS version 27; where the p-value < 0.05 was considered statistically significant.

**Results:**

A total of 344 patients were enrolled, with a male predominance (57.6%) and a majority (58.4%) aged over 18 years. The common clinical indications for antibiotic prescriptions were gastrointestinal infections (28.5%) followed by Respiratory tract (upper and lower) infections (27.4%). The antibiotics were mainly indicated for therapeutic purpose (59.3%). The antibiotic use prevalence was 85.8% with high rate (59.0%) of patients were receive “Watch” group antibiotics mainly from cephalosporin class. Empirical therapy was the predominant treatment approach (95.3%), with ceftriaxone being the most frequently prescribed agent, accounting for 43.3% of all empirical antibiotic use. Surgical prophylaxis accounted for the majority (88%) of prophylactic antibiotic use. Notably, nearly three-quarters (74%) of these patients received prophylaxis for longer than the recommended 24-hours. Only 4.0% of patients receiving parenteral antibiotics were switched to oral therapy. Furthermore, 37.1% of prescriptions deviated from guideline recommendations or lacked documentation to assess adherence. The treatment approach was rarely evidence-based, with microbiological testing absent in 90% of cases. Of patients who had culture results, gram-negative bacteria (e.g., *E. coli*,* Klebsiella spp.*,* Citrobacter spp.*) were a common bacterial isolates. Multivariable analysis showed that surgical procedures (AOR = 5.96) and peripheral catheters (AOR = 6.81) were significantly associated with increased antibiotic use.

**Conclusion:**

The findings reveal alarmingly high antibiotic utilization in Ethiopian hospitals, primarily driven by excessive empirical prescribing, insufficient microbiological testing, and inappropriate and prolonged surgical prophylaxis. These results highlight the urgent need for multifaceted interventions, including strengthening diagnostic capabilities to support evidence-based prescribing, implementing robust antimicrobial stewardship programs with regular ward-level audits, and ensuring strict adherence to treatment guidelines.

**Supplementary Information:**

The online version contains supplementary material available at 10.1186/s12879-026-12775-z.

## Background

Antimicrobial resistance (AMR) is a pressing global public health crisis, threatening the efficacy of antimicrobial agents and undermining decades of progress in combating infectious diseases. While AMR impacts both high- and low-income countries, the burden is disproportionately higher in low-resource settings, where weak health systems and a high prevalence of infectious diseases exacerbate the problem [1]. Of the total estimated 10 million annual deaths attributable to AMR by 2050, 4.1 million will be from Africa [2].

Patients infected with multidrug-resistant bacteria are at a significantly higher risk of severe complications, poor clinical outcomes, and mortality. These infections also demand more healthcare resources compared to those caused by non-resistant strains, placing additional strain on the already fragile health systems. Beyond the immediate health impacts, AMR imposes a substantial economic burden. When first-line treatments fail due to resistance, healthcare providers are forced to rely on more expensive, less accessible therapies, often requiring prolonged hospital stays and intensive care. This might not only increase healthcare costs but also could exacerbate the economic challenges faced by individuals, healthcare systems, and societies at large [3].

The global AMR crisis is driven by a combination of factors, including the over prescription and indiscriminate use of antimicrobials by healthcare providers, patients’ failure to complete prescribed treatment courses, and the widespread availability of substandard and falsified (SF) medicines. Inappropriate dosages, sub-optimal therapies, incorrect prescriptions, and inadequate infection prevention and control measures in healthcare settings further contribute to the problem. Broader societal issues, such as poor hygiene and sanitation, also play a significant role in fueling the spread of resistant infections. These interconnected failures have created a perfect storm, propelling AMR to the forefront of global health emergencies and necessitating urgent, coordinated action to avert the onset of a post-antibiotic era [1, 4, 5].

Continuous monitoring and evaluation of antimicrobial use, as recommended by the World Health Organization (WHO), are critical strategies for improving antibiotic prescribing practices, ensuring the ongoing efficacy of antimicrobials, and controlling resistance [6, 7]. Previous studies in Ethiopia have shed light on critical issues such as widespread antibiotic misuse and overuse, the alarming prevalence of resistant bacterial strains, and significant non-adherence to both global and national antibiotic treatment guidelines [7–11] as well as emergence of microbes that are resistant against locally available antibiotics including carbapenems [11–13].

In response to this growing threat, the Ethiopian government has adopted the WHO’s Global Action Plan (GAP) on AMR and implemented strategies such as Antimicrobial Stewardship Programs (ASPs) to combat resistance. However, there remains a lack of comprehensive national data on antibiotic utilization patterns, prevention of healthcare-associated infections (HAIs), and the quality of prescribing practice [13]. This study, therefore, aims to address this gap by providing a multicenter point-prevalence survey of antibiotic use in selected Ethiopian hospitals, offering critical insights to inform future stewardship efforts and policy interventions.

## Materials and methods

A cross-sectional study was conducted in August 2023 across Ethiopia’s three-tier public healthcare system. Using the WHO’s Point Prevalence Survey methodology for antibiotic use, four public hospitals were purposively selected based on their location and service catchment areas: Jimma Medical Center (tertiary), Agero General Hospital, Seka Primary Hospital, and Nada Primary Hospital. The selection aligned with the Ethiopian Ministry of Health’s strategic priorities for implementing and strengthening antimicrobial stewardship programs.

### Inclusion and exclusion criteria

The inclusion criteria were applied sequentially in a stepwise manner: first to hospitals, then to eligible wards within those hospitals, followed by patients admitted to the selected wards, and finally to the antibiotics prescribed or dispensed to those patients, in accordance with the WHO Point Prevalence Survey (PPS) methodology for LMICs version 1.1 [6]. Eligible participants included (1) Hospitalized patients with complete medical records admitted to acute care wards (pediatric medical, neonatal ICU, adult medical, adult surgical, gynecology, Pediatric high risk ward, orthopedics, and oncology) before or at 08:00 on survey day; (2) Neonates born before 08:00 (recorded separately from mothers); and (3) Active systemic (oral/parenteral) antibiotics at 08:00 (only antibiotic active at 08:00 recorded if changed later).

Accordingly, the exclusion criteria were: (1) Non-acute wards (long-term care, emergency departments, day surgery/dialysis units); and (2) Patients discharged before 08:00 (including those awaiting transport/same-day discharge).

### Outcome variables

The primary outcome was the prevalence antibiotic use while drivers (i.e. Patterns of antibiotic use, Quality indicators of antibiotic prescribing and associated factors of antibiotic use) were secondary outcomes.

### Sample size determination and sampling technique

The WHO Point Prevalence Survey (PPS) methodology was used for determination of sample size. Accordingly, the three hospitals (Agero general hospital, Seka and Nada primary hospitals) had fewer than 500 inpatient beds; and therefore, all eligible patients in their wards were included. On the other hand, Jimma Medical Center, with more than 800 inpatient beds, was sampled according to WHO PPS methodology [6], whereby one out of every three patients per ward was selected. As a result, formal sample size calculation was not required, as the number of bed spaces in each hospital was known prior to the survey. If a selected patient or medical record was unavailable, the next eligible record was included. This approach was consistently applied across all wards to ensure strict adherence to the predefined study procedures.

### Data collection and management

Data was collected using Open Data Kit (ODK) software (ODK, Seattle, WA, USA). The WHO PPS methodology questionnaire was utilized to collect data on antibiotic use and prescribing patterns among in-patients in the study hospitals [6]. This included information about (i) the hospital, (ii) ward, (iii) patient, (iv) indications, and (v) antibiotic use and microbiology data. Microbiology data for culture and antibiotic susceptibility test (AST) included blood, urine, wound/pus/discharge, stool, sputum/respiratory samples, sterile fluids including cerebrospinal fluid, peritoneal fluid, and synovial fluid.

**Fig. 1:**
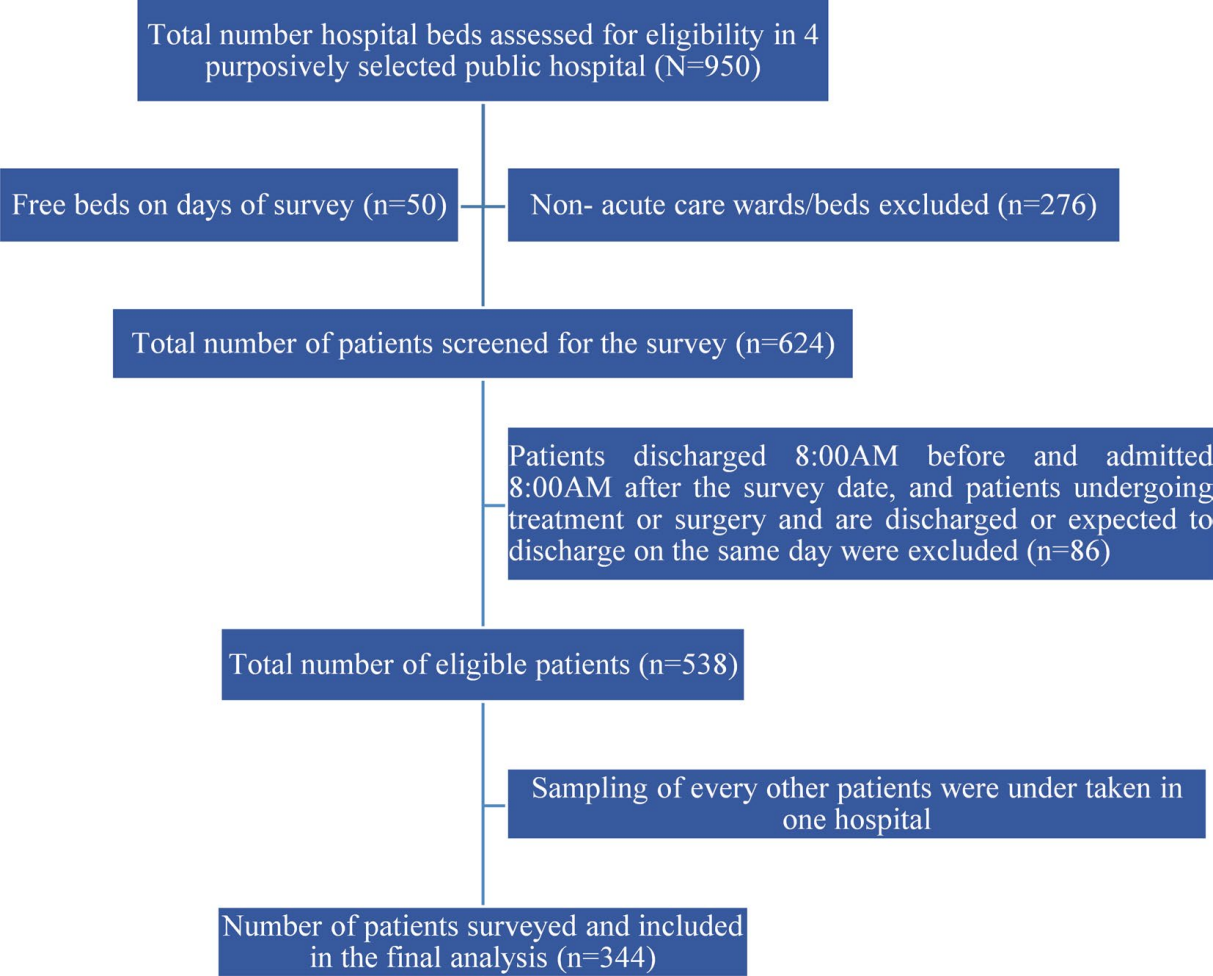
Diagrammatic scheme of study participant recruitment process

Data collection was executed by four data collectors who were specifically trained for this purpose and the team visited each hospital for a period of two days translating into an 8-day data collection period. The trainers were experts in conducting PPS in Ethiopia. The training was done for a period of three days to ensure that the data collectors understood the need to collect complete and good quality data. ODK accounts were opened for all the data collectors and testing of data entry was done on day-two and -three of the training. Data collectors arrived at each ward by 07:30 to review medical records, medication charts, and nursing notes to prevent missing of patient data. After conducting the PPS, a meeting was held with the hospital management and staff where the findings were disseminated, and recommendations were provided (Fig. 1).

### Data analysis

The collected data were extracted from ODK and exported to Microsoft Excel 2013 (Microsoft Corporation, Redmond, WA, USA). Data analysis was performed using IBM SPSS version 27.0 (IBM Corp., Armonk, NY, USA). Descriptive statistics were performed for hospital and ward demographic characteristics, patient data, indication, and antibiotic prescribing patterns and the results were presented in tables and charts as frequencies and percentages. Categorical variables with “Unknown” or missing values were retained as a separate category in descriptive analysis and were excluded from relevant sub-analyses.

To determine the prevalence of antibiotic use, the denominator was set to be the number of patients who met the inclusion criteria and were included in the survey. The numerator was the number of recruited patients who were currently on antibiotic treatment on the day of the survey. Prescribing compliance was assessed using the Ethiopia Standard Treatment Guidelines (STGs) [14].

## Results

### Patient demographics and clinical characteristics

A total of 344 patients participated in the study, with 58% of the participants were greater 18 years. Among the participants, 57% were male, 31% had surgical intervention since admission. About 85% of patients had used antibiotics, and nearly half (47%) had two antibiotics counter during their stay at the hospital. Majority (67.1%) of patients cases were referral either from hospital of non-hospital health facilities (Table 1).

**Table 1:** Baseline characteristics of the study participants

Variables	Categories	*Frequency(n = 344)*	Percentage (%)
Sex	Male	198	57.6
	Female	146	42.4
Age	< 24 months	87	25.3
	2–5 years	21	5.9
	6–12 years	20	5.8
	13–17 years	16	4.7
	18–35 years	98	28.5
	> 35 years	103	29.9
Type of preterm for pre-term babies, *n* = 31	Late preterm	28	90.3
	Moderate preterm	1	3.2
	Very preterm	2	6.5
Surgery since admission	Yes	108	31.4
	No	236	68.6
Types of surgery	Major	12	3.5
	Minor	96	27.9
Central vascular catheter	Yes	5	1.5
	No	339	98.5
Urinary Catheter	Yes	26	7.6
	No	318	92.4
Peripheral Venous Catheter	Yes	274	79.7
	No	70	20.3
Endotracheal intubation	Yes	56	16.3
	No	288	83.7
Antibiotic treatment	Yes	295	85.8
	No	49	14.2
Number of antibiotic counter	One	104	30.4
	Two	163	47.4
	Three	22	6.4
	Four	4	1.2
	Five	1	0.3
	Six	1	0.3
Comorbidities			
Malaria status	Yes	21	6.1
	No	149	43.3
	Unknown	174	50.6
TB status	Yes	12	3.5
	No	109	31.7
	Unknown	223	64.8
HIV status	Positive	4	1.2
	Negative	138	40.1
	Unknown	202	58.7
Transferred from Hospital	Yes	114	33.1
	No	227	60
	Unknown	3	0.9
Transferred from non-Hospital	Yes	117	34
	No	219	63.7
	Unknown	8	2.3
Hospitalization in past 90-days	Yes	78	22.7
	No	248	72.1
	Unknown	18	5.2

TB: Tuberculosis; HIV: Human Immune Deficiency Virus

### Hospital and wards categorization

Out of four hospital included, majority 194 (56.4%) of the patients were from Jimma Medical Center (JMC). Adult medical wards (24.1%) and surgical wards (18.9%) were leading wards from which the study participants were included in the study (S1 Table).

### Indication for antibiotics

Of the 295 patients who received antibiotics, nearly half (49.2%) were for community-acquired infections; while from 84 patients receiving antibiotic prophylaxis, 74 (88.1%) was for surgical indications. Additionally, among 175 patients treated for a specific bacterial infection, the most common diagnoses were gastrointestinal (28.5%) and respiratory tract (27.5%) infections (Table 2).

**Table 2:** Indications for antibiotics and types of infections

Variables	Category	Frequency (*N*)	Percentage (%)
Indications of antibiotics	Community acquired infections	145	49.2
	Hospital acquired infections	30	10.2
	Surgical prophylaxis	74	25.0
	Medical prophylaxis	10	3.4
	Not specified	36	12.2
Types of infections based on organ systems affected (*n* = 175)	Gastrointestinal infection	50	28.5
	Respiratory tract(upper and lower) infections	48	27.4
	Bone and joint infections	26	14.8
	Gynecological infection	14	8.0
	Systemic infections	12	6.8
	Central nerve system infections	10	5.7
	Urinary tract infection	10	5.7
	Cardiovascular infections	1	0.5
	Others*	4	2.2
Prophylaxis(*n* = 84)	Surgical prophylaxis	74	88.1
	Medical prophylaxis	10	11.9
Duration of surgical prophylaxis (*n* = 74)	Single dose	11	14.9
	Multi-dose in one day	8	10.8
	Multi-dose in more than one day	55	74.3
Surgical prophylaxis site	Central nervous system	1	1.4
	Otolaryngological procedures	4	5.4
	Gynecology & obstetrics	25	33.8
	Respiratory tract	1	1.4
	Skin, soft tissue, bone and joint	21	28.4
	Gastrointestinal tract	14	18.9
	Urinary tract	8	10.8

Others*:-Febrile neutropenia, cystic fibrosis, sexually transmitted infection, completely undefined site

### Antibiotics prescribing pattern by aware classification

According to the WHO AWaRe classification, 26.6% (92/344) of patients were prescribed antibiotics from “Access” groups: metronidazole (40), gentamicin (20), ampicillin (10), doxycycline (6), cephalexin (4), amoxicillin (2), Benzathine penicillin (2), trimethoprim-sulfamethoxazole (4), amoxicillin clavulanic acid (2) and clindamycin (2). Meanwhile, 59.0% (203/344) of patients received “Watch” group antibiotics: azithromycin (14), ceftriaxone (128), ciprofloxacin (6), ceftazidime (19), meropenem (2) and vancomycin (34) (Table 3).

**Table 3:** Antibiotics prescribing pattern by aware classification of antibiotics

Types of antibiotics used	Class of antibiotic	ATC code	Hospital level				
			JMC (N = 194), n (%)	AGH (N = 67), n (%)	SPH (N = 42) n (%)	NPH (N = 41) n (%)	AWaRe classification
Ceftriaxone	Cephalosporin	J01DD04	70(36.1)	23(34.3)	17(40.5)	18(43.9)	Watch
Benzathine benzylpenicillin	Penicillin	J01CE08	2(1.0)	0	0	0	Access
Cefalexin	Cephalosporin	J01DB01	1(0.5)	0	2(4.8)	1(2.4)	Access
Gentamicin	Aminoglycoside	J01GB03	9(4.6)	8(12.0)	1(2.4)	2(4.9)	Access
Ampicillin	Penicillin	J01CA01	4(2.1)	4(6.0)	0	2(4.9)	Access
Sulfamethoxazole/trimethoprim	Sulfonamide-trimethoprim-combinations	J01EE03	2(1.0)	0	2(4.8)	0	Access
Meropenem	Carbapenems	J01DH02	1(0.5)	1(1.5)	0	0	Watch
Clindamycin	Lincosamides	J01FF01	0	0	2(2.8)	0	Access
Amoxicillin	Penicillin	J01CA04	2(1.0)	1(1.5)	0	0	Access
Metronidazole	Imidazole	J01XD01 and P01AB01	24(12.4)	6(9.0)	5(16.7)	5(12.2)	Access
Azithromycin	Macrolide	J01FA10	13(6.7)	0	0	12(29.3)	Watch
Ceftazidime	Cephalosporin	J01DD02	13(6.7)	3(4.5)	2(4.8)	1(2.4)	Watch
Vancomycin	Glycopeptide	J01XA01 and A07AA09	21(10.8)	8(12)	3(7.1)	2(4.9)	Watch
Ciprofloxacin	Fluoroquinolone		6(3.1)	0	0	0	Watch
Doxycycline	Tetracycline	J01MA02	6(3.1)	0	0	0	Access
Amoxicillin/ Clavulanic acid	Beta-lactam/beta-lactamase-inhibit	J01CR02	1(0.5)	0	1(2.4)	0	Access

JMC-Jimma Medical Center, AGH-Agero General Hospital, SPH-Seka Primary Hospital, NPH-Neda Primary Hospital

### Antibiotics use prevalence

Two hundred ninety-five (85.8%) patients had receive antibiotics prescription, with Seka Primary Hospital counted highest rate (92.9%, 39/42) of antibiotic use. Similarly prescription rate of antibiotics was differ across hospitals units, with pediatric high-risk wards (95.5%, 43/45) and neonatal intensive care units (91.9%, 34/37) were among units with highest rate of antibiotics consumption (S2 Table).

### Types of antibiotics prescriptions by wards

Antibiotic consumption rates and types varied across different clinical unit. Ceftriaxone use was notably high in Oncology (60%; 3/5) and the Pediatric High-Risk Ward (46.7%; 21/45). Similarly, metronidazole was commonly prescribed in Oncology (20%; 1/5) and the Neonatal Intensive Care Unit (16.2%; 6/37) (S3 Table).

### Antibiotics prescription by clinical indication

Antibiotics were prescribed mainly for therapeutic or prophylaxis reasons. Community acquired infections were main reasons for therapeutic use of antibiotics, with ceftriaxone (46.9%, 68/145) and metronidazole (14.5%, 21/145) were the predominant antibiotics used for this clinical indication. Similarly preoperative antibiotics was also common reason, with ceftriaxone (35.1%, 26/74) and metronidazole (16.2%, 12/74) were highly prescribed for this case (S4 Table).

### Quality indicators for antibiotics use

A survey of all hospitals revealed a high rate of parenteral antibiotic prescribing. Likewise, there were poor antibiotic stewardship practices, evidenced by widespread non-compliance to guideline for prescribing antibiotics, a heavy reliance on empirical treatment, and an underutilization of microbiological testing (Table 4).

**Table 4:** Quality indicators for antibiotics use

Variable	Category	JMC (N = 194) n (%)	AGH (N = 67) n, (%)	SPH (N = 42), n (%)	NPH (N = 41), n (%)
Prescriber	General practitioner	169(87.1)	52(77.6)	42(100.0)	18(43.9)
	Specialist practitioner	25(12.9)	15(22.3)	0	23(56.1)
Administration Route	parenteral	162(85.3)	65(97)	34(81.0)	37(90.2)
	Oral	32(14.7)	2(3.0))	8(19.0)	4(9.8)
Parenteral	Intermittent intravenous	120(61.9)	36(53.7)	25(59.5)	28(68.3)
	Continuous intravenous	27(13.9)	22(32.8)	2(4.8)	4(9.8)
	Extended intravenous	12(6.2)	7(10.4)	7(16.7)	5(12.2)
	Intramuscular	35(18.0)	2(3.0)	8(19.1)	4(9.7)
Switched to oral	Yes	4(2.1)	0	6(14.3)	2(4.9)
	No	190(97.9)	67(100.0)	36(85.7)	39(95.1)
Number of missed doses	One	1(0.6)	2(3.0)	1(2.4)	2(4.9)
	Two	0	6(9.0)	1(2.4)	1(2.4)
	Three	0	0	2(4.9)	0
Reason for missed dose	Affordability	0	8(11.9)	4(9.5)	3(7.3)
	Unknown	1(0.6)	0	0	0
Guideline compliance	Yes	160(82.5)	52(77.6)	10(23.8)	29(70.7)
	No	22(11.3)	13(19.4)	32(76.2)	11(26.8)
	NA*	4(2.1)	2(3.0)	0	0
	NI*	8(4.1)	0	0	1(2.4)
Treatment types	Empirical therapy	186(95.9)	66(98.5)	38(90.5)	38(92.7)
	Targeted therapy	8(4.1)	1(1.5)	4(9.5)	3(7.3)
Specimen taken*	Yes	34(17.5)	0	0	0
	No	160(82.5)	67(100.0)	42(100.0)	41(100.0)
Types of specimen (*n* = 34)	Blood	14(41.2)	0	0	0
	Sputum/respiratory sample	7(20.6)	0	0	0
	Sterile fluid	6(17.6)	0	0	0
	Wound	5(14.7)	0	0	0
	Urine	2(5.9)	0	0	0
Culture report (*n* = 34)	Positive	18(52.9	0	0	0
	Negative	16(47.1)	0	0	0
Culture report positive**(*n* = 18)	Gram –ve	14(77.7)	0	0	0
	Gram +ve	4(22.3)	0	0	0
Antibiotic susceptibility test	Yes	2(11.1)	0	0	0
	No	16(88.9)	0	0	0

*NA: Not assessable because of the absence of any guidelines; NI: No information because of incomplete patient history; JMC-Jimma Medical Center, AGH-Agero General Hospital, SPH-Seka Primary Hospital, NPH-Neda Primary Hospital

*Specimen taken: any microbiological data ordered before or after prescription of antibiotic

**Culture positivity: proportion of patients with positive culture results among those who had any microbiological specimen taken

Culture positivity: was calculated as the proportion of patients with positive culture result among those who had any microbiological specimen taken

### Microbiological test

Of eighteen culture report, 14 (77.7%) culture results were gram negative bacterial isolates. *Escherichia coli* (28.5%), *Klebsiella spp*. (14.3%) and *Citrobacter spp. (14.3%)* were the dominant gram-negative isolate (Table 5).

**Table 5:** Bacterial isolate of culture results

Micro organism		Frequency(*n*)	Percentage (%)
Gram-Positive(*n* = 4)	*Streptococcus agalactiae*	1	25.0
	*Streptococcus pneumoniae*	1	25.0
	*Staphylococcus aureus*	1	25.0
	*Coagulase-negative staphylococci*	1	25.0
Gram-Negative(*n* = 14)	*Escherichia coli*	4	28.5
	*Klebsiella spp.*	2	14.3
	*Citrobacter spp.*	2	14.3
	*Proteus vulgaris*	1	7.1
	*Serratia spp.*	1	7.1
	*Acinetobacter*	1	7.1
	*Klebsiella pneumoniae*	1	7.1
	*Proteus mirabilis*	1	7.1
	*Aggregatibacter actinomycetemcomitans*	1	7.1

### Antimicrobial susceptibility results of bacterial isolates

Antibiotics susceptibility test was rarely (only two patients) checked for identified bacterial isolates. Gram-negative bacilli exhibited extensive resistance to multiple classes of antibiotics. *Escherichia coli* was the most resistant strain, resistant to eight antibiotics, followed by *Proteus mirabilis*,* Citrobacter spp.*,* and Serratia*, each resistant to six antibiotics. Among Gram-positive bacteria, *methicillin-resistant Staphylococcus aureus* (MRSA) was identified, demonstrating resistance to four antibiotics, including multiple beta-lactams (ampicillin and penicillin) (S5 Table).

### Factors associated with antibiotic use

On multivariate logistic regressions having history of surgical procedure since admission [AOR = 5.96, 95%CI(2.28–15.62), *P* = 0.001], and having peripheral vascular catheter [AOR = 6.81,CI(3.35–13.85), *P* = 0.001] were predictors of antibiotic use; whereas admitted to adult surgical ward [AOR = 0.06, 95%CI (0.01–0.34), *P* = 0.001], Gynecology & Obstetrics ward [AOR = 0.12, 95%CI (0.02–0.67), *P* = 0.02], Orthopedic ward [AOR = 0.06, 95%CI (0.01–0.41), *P* = 0.004], protective for antibiotic use (Table 6).

**Table 6:** Factors associated with antibiotics use

Variables	Categories	Patient on antibiotics		p-value	COR, 95%CI	P-value	AOR, 95%CI
		Yes, n (%)	No, n (%)				
Ward types*	PMW	36(12.2)	4(8.2)	0.33	0.42(0.07–2.42	0.19	0.3(0.05–1.87)
	NICU	34(11.5)	3(6.1)	0.49	0.53(0.08–3.34)	0.34	0.39(0.06–2.64)
	AMW	72(24.4)	11(22.4)	0.13	0.30(0.06–1.44	0.15	0.31(0.06–1.52)
	ASW	54(18.3)	13(26.5)	0.04	0.19(0.04–0.90)	0.001	0.06(0.01–0.34)*
	GOB	27(9.2)	8(16.4)	0.03	0.16(0.03–0.79	0.02	0.12(0.02–0.67)*
	ORTHO	25(8.5)	7(14.3)	0.03	0.17(0.03–0.86)	0.004	0.06(0.009-0.40)*
	ONCO	4(1.4)	1(2.0)	0.21	0.19(0.01–2.53)	0.09	0.10(0.007–1.48)
	PHRW	43(14.6)	2(1.4)	1			
Gender	Female	122(41.4)	24(49.0)	0.391	0.735(0.401–1.34)		
	Male	173(51.0)	25(58.6)	1			
Surgery since admission	Yes	97(32.9)	11(10.2)	0.148	0.591(0.29–1.21)*	< 0.001	5.96(2.28–15.62)**
	No	198(67.1)	38(77.6)	1			
Peripheral catheterization status	Yes	252(85.4)	22(44.9)	< 0.001	0.139(0.073–0.266)*	< 0.001	6.81(3.35–13.85)**
	No	43(14.6)	27(55.1)	1			
Prescriber type	General practitioner	238(80.7)	43(87.8)	0.24	0.583(0.236–1.44)*		
	Specialist practitioner	57(19.3)	6(12.2)	1			

PMW: Pediatric Medical Ward; NICU: Neonatal Intensive Care Unit; AMW: Adult Medical Ward; ASW: Adult Surgical Ward; GOB: Gyneobstetrics Ward; Orthopedic Ward; ONCO: Oncology; PHRW: Pediatric High Risk ward; COR: Crude odds Ratio; AOR: Adjusted Odds Ratio

## Discussion

This multicenter point-prevalence survey across three tiers of Ethiopia’s public healthcare system reveals a high prevalence of antibiotic use (85.8%), characterized by a heavy reliance on empirical therapy, excessive use of WHO ‘Watch’ group antibiotics (particularly ceftriaxone), and poor adherence to several quality indicators including prolonged surgical prophylaxis and a low rate of intravenous-to-oral conversion. These practices occurred within a context of severely limited microbiological diagnostic support. The findings align with patterns observed in other low-resource settings and underscore systemic challenges in antimicrobial stewardship.

The study shows high rate of parenteral antibiotic use, with 95% of the patients received these antibiotics for empirical coverage of suspected bacterial infections. While life-saving in emergencies, this heavy reliance on empirical over definitive is a primary driver of antimicrobial resistance (AMR) [15]. The root cause is a critical diagnostic gap as evidenced in this study and lack of guideline compliance: a simple lack of testing tools at primary levels and a failure to consistently utilize them even where they exist like tertiary hospital; this practice urges need of stewardship diagnostic tools to shift into evidence based treatment approach for better outcomes of the patients [16].

Our study identified approximately two third (74.3%) of patients who had received preoperative antibiotics, used for longer than 24 h, while guidelines advise discontinuing preoperative antibiotics after 24 h if no infection is present [17]. The problem on preoperative antibiotics is not limited to use for longer duration, but also frequent dose of antibiotics with in the recommend duration of prophylaxis, suggesting potential contributing factors for antimicrobial resistance which hinder to achieve the desired patients treatment outcomes.

Furthermore, the switch from IV to oral antibiotics was strikingly rare occurring only in 3.5% of eligible patients; despite guideline recommend switching of antibiotic when there is no clear benefits for patient [18]. This prescribing practices had observed across all study sites in this study; underscores the urgent need to strengthen and fully implement antimicrobial stewardship programs.

Our data showed a systemic overuse of antibiotics, with an 85.8% prevalence rate, with slightly higher at secondary and primary hospitals. This pattern of empirical prescribing, aligned with trends in other Sub-Saharan Africa, notably in Eswatini (88.2%) [19] and Nigeria (81.3%) [20] occurs in a diagnostic vacuum: as only 9.8% of patients had culture results to guide therapy in our study. However, it is markedly higher than prevalence reported in previous studies from Ethiopia (63%-65%) [10, 21], Zambia (59.0%) [22], Sierra Leone (73.3%) [23], Thailand (53.0%) [24], and Tanzania (47%) [25]. This discrepancy likely stems from types of hospitals included as our study included primary and secondary hospitals with less established stewardship programs, unlike prior studies confined to tertiary hospitals where prescribing protocols are typically more robust.

The utilization pattern of antibiotics is also different across different units/wards of the hospitals. This study reveals higher prescribing pattern of antibiotics at Neonatal Intensive Care Unit (NICU) (91.9%) and Pediatric High Risk Ward (PHRW) (95.5%). Our finding is consistent with the finding of the studies from Sierra Leone (85.7% %, pediatric ward) [23], Thailand (53.3%, NICU) [24], and Kenya (59%, pediatric ward) [26] which were reported higher rate of antibiotics utilization pattern at these wards as compared to other wards. However, it is contrary to studies from United Arab Emirates (higher usage at gynecology and surgical wards) [27] and Ethiopia (higher usage at adult ICUs and pediatric emergency ward) [10]. This inconsistency may be attributed to two key factors: a higher rate of non-guideline compliance in antibiotic prescribing in our settings as compared to the stricter adherence to guideline at UAE, and the absence of patients from adult ICUs and pediatric emergency wards in our study as compared to prior study from Ethiopia.

Prescribing patterns were dominated by “Watch” group antibiotics, notably from cephalosporin class of antibiotic, with ceftriaxone a predominant. Nearly one of five patients were received ceftriaxone prescription for therapeutic management of community-acquired infections. Such empirical use of ceftriaxone for unconfirmed cases of bacterial infections was similarly reported by prior studies [5, 10, 21, 28–30], reflects its a wide spectrum of activity, and low toxicity causing strong selection pressure by clinician. Similarly, from “access” group metronidazole is highly prescribed by clinician mainly for therapeutics purpose and surgical prophylaxis, reflects a syndrome-based empirical approach, without microbiological confirmation of anaerobic involvement. This prescription pattern is concerning as documented in our study, of eighteen patients with positive culture results, three fourth of them had gram negative bacterial bacilli gross which were resistant to commonly prescribed antibiotics. This process is vicious cycle: the lack of culture results forces empirical therapy, whose widespread and frequent use fuels resistance, making subsequent empirical decisions even more challenging and dangerous [31].

Microbiologic test was rarely ordered for patients suspected with bacterial infections due to either lack of clear protocol and diagnostic tools at these health facilities. Only one hospital (Jimma medical center) had microbiological lab for confirming bacterial infections among suspected patients to support the initiation of antibiotics. Even in this hospital, only 34 patients had culture result, of which nearly 53% had bacterial growth. About three four of bacterial growth were gram negative bacterial isolates, with Escherichia coli (22.2%) and Klebsiella spp. (16.7%) were a frequent isolates. The predomince of gram negative bacterial isolates were similar to previous finding from Ethiopian studies [5, 10, 22].

Additionally, antibiotics susceptibility test is not routinely performed, unless there is suspected treatment failure for patients treated for bacterial infection. Only two patients have drug susceptibility test due to suspected treatment failure while they were on antibiotics. Their results had showed extensive multi-drug resistance; particularly E. coli was evidenced resistant to eight antibiotics, including “Watch” group agents like ceftriaxone, ciprofloxacin, and amoxicillin-clavulanic acid. This type of resistance pattern to commonly used antibiotic for empirical treatment like ceftriaxone is concerning, reflects the necessity of shifting from empirical treatment to targeted therapy to tacking the rapidly increasing risk antimicrobial resistance.

The associated factors of antibiotics use are: having surgical procedure since admission, peripheral vascular catheter in place, admitted to adult surgical ward, gynecology & obstetrics ward and orthopedic ward. Having surgical procedure history and peripheral vascular catheter are positively associated with antibiotics use; whereas admission to adult surgical ward, gynecology & obstetrics ward and orthopedic ward are negatively associated.

Patients who have undergone surgery are about six times more likely to receive antibiotics than those who have not, a finding consistent with previous studies [10, 32]. Similarly, Patients with a peripheral vascular catheter are about seven times more likely to receive antibiotics than those without vascular catheter, consistent with established evidence that breaches in the body’s natural barriers, such as those created by medical devices, significantly elevate infection risk, prompting antibiotic use for both prophylaxis and treatment [33–35].

The finding confirms that patients in adult surgical, gynecological, and orthopedic wards have lower odds of antibiotic prescription. Patients in the adult surgical ward, are 94% less likely to be on antibiotics as compared to patients in the pediatric high-risk ward, which similar to the finding of previous studies [36], reflects immature immune system (both innate and adaptive) between pediatric and adult or indication of admission to a Pediatric High-Risk Ward are often because of a suspected or confirmed infection. Similarly patients who were admitted to Gynecology & Obstetrics ward are 88% less likely to be on antibiotic; whereas patients who were admitted to orthopedic ward were 94% less likely to be on antibiotics.

Our study has several limitations. First, this study employed a WHO point-prevalence survey design, capturing only antibiotic therapies active at 08:00 on the survey day except antibiotics which were prescribed but not administered and antibiotics which have long dosing interval like Gentamicin which might be given in past 24 h. Antibiotics initiated later that day or short-course regimens completed before 08:00 were not recorded, potentially underestimating overall daily antibiotic use, particularly for single-dose or intermittent prophylactic therapies. Secondly, only purposively selected hospitals were included in the study. Therefore, the findings of this study may not be generalizable to all settings. Thirdly, this is point prevalence survey which show snapshot of antibiotic use pattern and lacks follow up of patients to capture long outcomes of patients such as length of hospital stay, duration of antibiotic therapy, both treatment and clinical outcomes of patients with bacterial infections. Fourth, it relied on available documentation, which may miss undocumented indications, comorbidities, or clinical rationale.

Fifth, the microbiological data reported is not representative of hospital population as it’s not routinely ordered for all patients. It mostly conducted for patients with severe diseases and those who failed first-line antibiotic therapy. However, with these limitation our study is the first multicentre study in Ethiopia containing the three tier hospitals to report antibiotic consumption rate and practice using a standardized WHO PPS methodology. This is useful for Stewardship Benchmarking that will in turn enhance identification of priority wards or hospitals for policymakers and other concerned bodies in strengthening ASP, optimizing antibiotics use and containing and preventing AMR.

## Conclusion

This multicenter survey discloses high and unsustainable rate of antibiotic use in Ethiopian hospitals, driven by excessive empirical prescribing, a critical lack of diagnostics, and inappropriate antibiotic prophylaxis. Excessive use of Watch-group antibiotics, particularly ceftriaxone, without microbiologic justification has directly selected for highly resistant gram-negative pathogens, as evidenced by the isolates of *E. coli* and *Klebsiella spp*. resistant to multiple first-line agents. To prevent this problems comprehensive interventions are imperative: improving microbiologic lab capabilities to facilitate, a shift from empirical to targeted therapy and strengthen Antimicrobial Stewardship Programs (ASPs) at both facilities and units.

## Supplementary Information

Below is the link to the electronic supplementary material.

Supplementary Material 1

## Data Availability

Reviewers or editors who wish to obtain the data and materials from this study can request them from the corresponding author upon reasonable request.
