# Supplementary material for: A multicenter point-prevalence survey of antibiotic utilization patterns in Ethiopia: implications for strengthening stewardship programs
Source: BMC Infect Dis. 2026 Feb 3;26:483. doi: 10.1186/s12879-026-12775-z (PMC12958573; doi:10.1186/s12879-026-12775-z)
Supplement: Supplementary file 1 — Supplementary Material 1 [file 12879_2026_12775_MOESM1_ESM.docx]

**S1 Table: Distribution of patients’ participation by hospital and ward**

| **Hospital name** | ***antibiotic use prevalence by hospital*** | ***Antibiotic Use prevalence by ward*** | | | | | | | |
| --- | --- | --- | --- | --- | --- | --- | --- | --- | --- |
|  |  | **PMW** | **ONCO** | **PHRW** | **NICU** | **AMW** | **ASW** | **GOB** | **ORTHO** |
| JMC | 82.00(159/194 | 90(36/40) | 80(4/5) | - | 72.7(8/11) | 82.6(38/46) | 86.5 (32/37) | 69.6(16/23) | 78.1(25/32) |
| AGH | 89.60(60/67) | - | - | 92.3(12/13) | 100(11/11) | 100(20/20) | 66.7(10/15) | 87.5(7/8) | - |
| SPH | 92.90(39/42) | - | - | 100(11/11) | 100(6/6) | 91.7(11/12) | 77.8(7/9) | 100(4/4) | - |
| NPH | 90.20(37/41) | - | - | 95.2(20/21) | 100(9/9) | 60(3/5) | 83.3(5/6) | - | - |
| Overall | 85.8(295/344) | 90(36/40) | 80(4/5) | 95.5(43/45) | 91.9(34/37) | 86.7(72/83) | 80(52/65) | 77.1(27/35) | 78.1(25/32) |

**S2 Table: Prevalence of Antibiotic Use by Hospital and Ward among Hospitalized Patients**

PMW: Pediatric Medical Ward; ONCO: Oncology Ward; PHRW: Pediatric High Risk Ward; NICU: Neonatal Intensive Care Unit; ASW: Adult Surgical Ward; AICU: Adult Intensive Care Unit; GOB: Gyneobstristic Ward; ORTHO: Orthopedic Ward

S3 **Table: Types of antibiotics prescriptions by wards**

PMW: Pediatric Medical Ward; NICU: Neonatal Intensive Care Unit; AMW: Adult Medical Ward; ASW: Adult Surgical Ward; GOB: Gyneobstristic Ward; Orthopedic Ward; ONCO: Oncology; PHRW: Pediatric High Risk ward

| ***Types of antibiotics used*** | ***ATC code*** | ***Ward types*** | | | | | | | |
| --- | --- | --- | --- | --- | --- | --- | --- | --- | --- |
|  |  | **PMW (N=40), n (%)** | **NICU (N=37), n (%)** | **AMW (N=83), n (%)** | **ASW (N=67), n (%)** | **GOB (N=35), n (%)** | **ORTHO (N=32), n (%)** | **ONCO (N=5), n (%)** | **PHRW (N=45), n (%)** |
| Ceftriaxone | J01DD04 | 18(45.0) | 16(43.2) | 31(37.3) | 24(35.8) | 7(20.0) | 8(25.0) | 3(60.0) | 21(46.7) |
| Benzathine benzylpenicillin | J01CE08 | 1(2.5) | 0 | 0 | 0 | 1(2.9) | 0 | 0 | 0 |
| Cefalexin | J01DB01 | 1(2.5) | 0 | 2(2.4) | 0 | 0 | 0 | 0 | 1(2.2) |
| Gentamicin | J01GB03 | 0 | 3(8.1) | 1(1.2) | 3(4.5) | 4(11.4) | 5(15.6) | 0 | 4(8.9) |
| Ampicillin | J01CA01 | 0 | 2(5.4) | 1(1.2) | 1(1.5) | 4(11.4) | 1(3.1) | 0 | 1(2.2) |
| Sulfamethoxazole/trimethoprim | J01EE03 | 0 | 0 | 1(1.2) | 0 | 0 | 2(6.2) | 0 | 1(2.2) |
| Meropenem | J01DH02 | 0 | 0 | 0 | 0 | 0 | 1(3.1) | 0 | 1(2.2) |
| Clindamycin | J01FF01 | 0 | 0 | 1(1.2) | 1(1.5) | 0 | 0 | 0 | 0 |
| Amoxicillin | J01CA04 | 0 | 0 | 1(1.2) | 1(1.5) | 1(2.9) | 0 | 0 | 0 |
| Metronidazole | J01XD01 and P01AB01 | 6(15.0) | 6(16.2) | 7(8.4) | 10(14.9) | 2(5.7) | 1(3.1) | 1(20.0) | 7(15.6) |
| Azithromycin | J01FA10 | 2(5.0) | 0 | 6(7.2) | 5(7.5) | 0 | 0 | 0 | 1(2.2) |
| Ceftazidime | J01DD02 | 1(2.5) | 0 | 7(8.4) | 3(4.5) | 4(11.4) | 3(9.4) | 0 | 1(2.2) |
| Vancomycin | J01XA01 and A07AA09 | 2(5.0) | 3(8.1) | 11(13.3) | 7(10.4) | 6(17.0) | 7(21.9) | 0 | 1(2.2) |
| Ciprofloxacin |  | 3(7.5) | 0 | 2(2.4) | 0 | 1(2.9) | 0 | 0 | 0 |
| Doxycycline | J01MA02 | 1(2.5) | 0 | 3(3.6) | 2(3.0) | 0 | 0 | 0 | 0 |
| Amoxicillin/ Clavulanic acid | J01CR02 | 0 | 1(2.7) | 0 | 1(1.5) | 0 | 0 | 0 | 0 |

| ***Types of antibiotics*** | ***ATC code*** | ***Therapeutic*** | | ***Prophylaxis*** | | ***Indication not specified (N=36), n (%)*** |
| --- | --- | --- | --- | --- | --- | --- |
|  |  | **CAIs (N=145), n (%)** | **HAIs (N=30), n (%)** | **MP (N=10), n (%)** | **SP (N=74), n (%)** |  |
| Ceftriaxone | J01DD04 | 68(46.9) | 12(40.0) | 4(40.0) | 26(35.1) | 15(41.7) |
| Benzathine benzylpenicillin | J01CE08 | 0 | 0 | 0 | 2(2.8) | 0 |
| Cefalexin | J01DB01 | 2(1.4) | 0 | 0 | 1(1.3) | 1(2.8) |
| Gentamicin | J01GB03 | 8(5.5) | 2(6.7) | 0 | 7(9.5) | 3(8.3) |
| Ampicillin | J01CA01 | 3(2.1) | 2(6.7) | 1(10.0) | 3(4.0) | 2(5.6) |
| Sulfamethoxazole/trimethoprim | J01EE03 | 0 | 0 | 0 | 2(2.8) | 1(2.8) |
| Meropenem | J01DH02 | 1(0.7) | 0 | 0 | 1(1.3) | 0 |
| Clindamycin | J01FF01 | 3(2.1) | 0 | 0 | 0 | 0 |
| Amoxicillin | J01CA04 | 1(0.7) | 0 | 0 | 0 | 1(2.8) |
| Metronidazole | J01XD01 and P01AB01 | 21(14.5) | 4(13.3) | 1(10.0) | 12(16.2) | 4(11.1) |
| Azithromycin | J01FA10 | 8(5.5) | 1(3.3) | 0 | 1(1.3) | 3(8.3) |
| Ceftazidime | J01DD02 | 7(4.8) | 6(20.0) | 1(10.0) | 5(6.8) | 1(2.8) |
| Vancomycin | J01XA01 and A07AA09 | 16(11.0) | 1(3.3) | 2(20.0) | 10(13.5) | 3(8.3) |
| Ciprofloxacin | J01MA02 | 2(1.4) | 2(6.7) | 1(10.0) | 1(1.3) | 1(2.8) |
| Doxycycline | J01AA02 | 4(2.7) | 0 | 0 | 2(2.8) | 1(2.8) |
| Amoxicillin/ Clavulanic acid | J01CR02 | 1(0.7) | 0 | 0 | 1(1.3) | 0 |

**S4 Table: Clinical indications of antibiotics use**

CAIs: Community acquired infections; HAIs: Hospital acquired infections

**S5 Table: Antimicrobial Susceptibility results of bacterial Isolates**

| ***Bacterial isolate*** | ***Antibiotics tested*** | | | | | | | | | | | | | | | | | | | |
| --- | --- | --- | --- | --- | --- | --- | --- | --- | --- | --- | --- | --- | --- | --- | --- | --- | --- | --- | --- | --- |
|  | AMP | CIP | GEN | PCN | TTC | CAF | ERY | CLI | CEF | TMX | MER | PIP | CEFO | CEFT | CEFU | DOXY | AMP/CLA | CEFE | TOB | VAN |
| *Proteus Vulgaris* |  | R | R |  |  | R |  |  |  |  |  |  | R | R |  |  |  |  |  |  |
| *Proteus Mirabilis* | R | R |  |  |  | R |  |  |  |  |  |  | R | R |  |  |  |  | R |  |
| *Klebsiella Pneumonia* |  |  |  |  |  |  |  |  |  |  |  |  |  |  |  |  | R |  |  | IM |
| *Klebsiella spp.* |  |  | S |  |  |  |  |  |  |  |  | S |  |  |  |  | S |  |  |  |
| *Staphylococcus aureus* | R |  |  | R | R |  |  |  | R |  |  |  |  |  |  |  |  |  |  |  |
| *Citrobacter spp.* |  | R | R |  |  |  |  |  | R | R |  |  | R | R |  |  |  |  |  |  |
| *Staphylococcus saprophyticus* |  |  |  |  |  |  |  |  |  |  |  |  |  |  |  | S |  |  |  | S |
| *Aggregatibacter actinomycetemcomitans* |  |  |  |  |  |  |  |  |  |  | R |  |  |  |  |  |  |  |  |  |
| *Escherichia coli* | R | R |  |  |  |  |  |  | R | R | S | R | R |  |  |  | R | R |  |  |
| *Streptococcus agalactiae* | R |  |  | R | R |  | R |  |  |  |  |  |  |  |  |  |  |  |  |  |
| *Serratia* |  | IM | R |  |  |  |  |  | R | R | R | IM |  | R | R |  |  |  |  |  |
| *Klebsiella ozaenae* |  |  | R |  |  |  |  |  | R | R | R | R |  |  | R |  |  |  |  |  |
| *Streptococcus pneumoniae* |  |  |  |  |  |  | R | R |  | R |  |  |  |  |  |  |  |  |  |  |

*AMP = Ampcillin, CIP = Ciprofloxacin, GEN=gentamycin, PCN=penicillin, TTC=tetracycline, CAF=chloramphenicol, ERY=erythromycin, CLI=clindamycin, CEF=ceftriaxone, TMX= Trimethoprim-sulphamethoxazole, MER=meropenem, PIP= piperacillin/tazobactam,CEFO= Cefotaxime,CEFT=ceftazidime,CEFU=Cefuroxime,DOX=Doxycycline, AMP/CLA=Amoxicillin-clavulanic acid,CEFE=Cefepime,TOB= Tobramycin,VAN= Vancomycin,S=sensitive,R=Resistant,IM=Intermediate*

| ***Variables*** | ***Categories*** | ***Frequency(n)*** | ***Percentage (%)*** |
| --- | --- | --- | --- |
| Hospital code | JUMC | 194 | 56.4 |
|  | AGH | 67 | 19.5 |
|  | SPH | 42 | 12.2 |
|  | NPH | 41 | 11.9 |
| Ward type | Adult Medical Ward | 83 | 24.1 |
|  | Adult Surgical Ward | 67 | 19.48 |
|  | Pediatric High-risk Wards | 45 | 13.1 |
|  | Pediatric Medical Ward | 40 | 11.6 |
|  | Neonatal Intensive Care Unit | 37 | 10.8 |
|  | Obstetrics/gynecology ward | 35 | 10.2 |
|  | Orthopedic ward | 32 | 9.3 |
|  | Oncology ward | 5 | 1.5 |

JMC-Jimma Medical Center, AGH-Agero General Hospital, SPH-Seka Primary Hospital, NPH-Neda Primary Hospital
